# Enhancing Visual Feedback Control through Early Fusion Deep Learning

**DOI:** 10.3390/e25101378

**Published:** 2023-09-25

**Authors:** Adrian-Paul Botezatu, Lavinia-Eugenia Ferariu, Adrian Burlacu

**Affiliations:** Faculty of Automatic Control and Computer Engineering, “Gheorghe Asachi” Technical University of Iasi, D. Mangeron 27, 700050 Iasi, Romania; adrian-paul.botezatu@academic.tuiasi.ro (A.-P.B.); lavinia-eugenia.ferariu@academic.tuiasi.ro (L.-E.F.)

**Keywords:** visual feedback control, convolutional neural network, early fusion, segmentation, feature points, image moments

## Abstract

A visual servoing system is a type of control system used in robotics that employs visual feedback to guide the movement of a robot or a camera to achieve a desired task. This problem is addressed using deep models that receive a visual representation of the current and desired scene, to compute the control input. The focus is on early fusion, which consists of using additional information integrated into the neural input array. In this context, we discuss how ready-to-use information can be directly obtained from the current and desired scenes, to facilitate the learning process. Inspired by some of the most effective traditional visual servoing techniques, we introduce early fusion based on image moments and provide an extensive analysis of approaches based on image moments, region-based segmentation, and feature points. These techniques are applied stand-alone or in combination, to allow obtaining maps with different levels of detail. The role of the extra maps is experimentally investigated for scenes with different layouts. The results show that early fusion facilitates a more accurate approximation of the linear and angular camera velocities, in order to control the movement of a 6-degree-of-freedom robot from a current configuration to a desired one. The best results were obtained for the extra maps providing details of low and medium levels.

## 1. Introduction

Visual feedback control is an important aspect of many modern applications, ranging from robotics to virtual reality. The ability to perceive and respond to visual information in real time is essential for achieving desired outcomes. The purpose of a visual feedback control architecture is to control a robotic system using information obtained from a visual sensor [1,2]. The visual sensor can be placed either on the robot (known as the “eye-in-hand” configuration [3]) or in another location in the workspace (known as the “eye-to-hand” configuration). This paper will examine the eye-in-hand configuration.

Visual feedback control can be achieved through various methods, such as classical visual servoing, which includes two popular methods, image-based visual servoing (IBVS) and pose-based visual servoing (PBVS) [3]. In IBVS, the control loop is driven by the error between the desired and current visual features, such as image coordinates, edges, corners, or image moments, while in PBVS, the control loop is driven by the error between the desired and current object pose. Despite their success in many applications, classical visual servoing methods have several limitations. They rely on hand-crafted features, predefined models, and linear control laws, which can result in poor performances under challenging conditions, such as occlusions, lighting changes, and cluttered environments [3].

Recent advances in deep learning have shown promise in enhancing visual feedback control. Instead of relying on hand-crafted features, deep learning techniques allow the system to learn and extract features directly from raw visual data. Convolutional neural networks (CNNs) have been shown to offer significant benefits when working with images, particularly in their ability to learn relevant features for a specific problem, without requiring predetermined feature extraction methods. To enable fast training, numerous neural architectures were built based on CNNs that were pre-trained for classification tasks, such as AlexNet [4], VGG-16 [5], or FlowNet [6]. In [7], Saxena et al. used FlowNet to perform the visual servoing task in various environments, without any prior knowledge of the camera parameters or scene geometry. The network predicts the camera’s pose by taking an input array that concatenates the images that specify the current and desired final scenes. The neural architectures presented by Bateux et al. [8] were derived from AlexNet and VGG16. They are capable of predicting the transformation that occurred in a camera through two images and customized to perform high-precision, robust, and real-time six degrees of freedom (DOF) positioning tasks by using visual feedback. The authors used a synthetic training dataset to support effective learning and improved robustness to illumination changes. In [9], the authors introduced DeFiNet, designed as a Siamese neural architecture. The features are extracted by two CNNs, which share neural parameters. The resulting features are passed to a regression block to predict the relative pose resulting from the current and target images that are captured by an eye-to-hand camera. Ribeiro et al. [10] compared three CNN-based architectures for grasping detection, where the neural network should provide a 3D pose, starting from two input images that describe the initial and final layouts of the scene. In the first architecture, a single branch is used, where the two input images are concatenated along the depth dimension to form the input array, and a single regression block generates all six outputs. The second model also uses the same input array but has two separate output branches for position and orientation. In contrast, the last CNN uses a separate feature extractor for each input image, concatenates the extracted features, and then uses a single regression block. Based on experimental results, the first model, which uses a single branch, yields the best performance. Regardless of the neural architectures or design of the learning techniques, some limitations arise from the inherent characteristics of data-driven methods. This includes the fact that the generalization capabilities of the deep model rely on the content of the training dataset, and important modifications of the visual elements captured in the scenes should involve retraining the model.

The technique of early fusion in neural networks involves incorporating additional information that is available to the input of the network. Since the method only extends the depth of the input array, without requesting the use of additional neural layers or connections, the approach does not involve significantly increased computational resources and could remain compatible with real-time scenarios [11,12]. This additional information can be obtained through various methods, such as segmentation [13], feature points [14], filtering, or utilizing information acquired from multiple sensors or sources to provide a more comprehensive understanding of the environment [15]. Another approach to data fusion is to integrate the new information into the hidden neural layers, a technique referred to as middle fusion. By integrating the supplementary data into the feature maps at different layers of the feature extractor, the extra information could potentially have a greater impact on global features. However, implementing middle fusion requires significant modifications to the neural architecture and may limit the transfer of learning from pre-trained models. The third possible approach means applying fusion at the decision level, which is the simplest approach and does not require modifications to the neural architecture. With this method, the supplementary data are not used during learning and are only incorporated at the decision-making stage.

The current work is set to evaluate multiple convolutional neural architectures proposed for a visual servoing task. This effectively integrates valuable supplementary knowledge and facilitates the transfer of learning from a CNN pre-trained for the image classification task, this being illustrated by comparing it with classical PBVS (pose-based visual servoing). To achieve this, the CNN architecture utilizes the early fusion method. The proposed template consists of exploiting traditional visual servoing techniques to produce additional useful input data for the deep models. In this regard, the extension of the input neural arrays is discussed for three relevant approaches: region-based segmentation, feature points, and image moments. These three approaches can offer different levels of detail from the initial and final scenes:

- The feature points can be considered as providing low-level information, as they give specific points in the image where single or multiple objects of interest are located;
- The regions indicated by segmentation can be considered as providing mid-level information, as they give a more general idea of the location and features of an object, by dividing the image into different areas;
- The image moments can be considered as providing high-level information, as they compute a summary of the distribution of pixel intensities in the image, which can be used to estimate the pose of the object, but also in decoupling the linear and angular camera velocities.

This paper investigates how traditional techniques could be effectively exploited by deep learning for the visual servoing task. In this regard, we discuss an early fusion approach for CNN-based visual servoing systems, which mixes the initial and final images with maps that illustrate additional information from these scenes. According to our knowledge, this is the first comprehensive analysis of additional ready-to-use information that can improve CNN’s ability to accurately perform the visual servoing task. The relevance of this extra information has been already validated by classic visual servoing techniques, however, the challenge consists of finding proper, simplified descriptions of the initial and final scenes, which could be useful for CNN. The maps should offer ready-to-use information to guide the extraction of features, for an effective computation of the linear and angular camera velocities for a 6 DOF robot. As all necessary information is available in the original images, the extra maps implicitly introduce data redundancy. The extra maps also require expanding the deep model with supplementary learnable parameters. As a consequence, an important aspect to analyze is what level of detail could be more helpful for CNN to make its training easier and more effective.

The main contributions of this work can be summarized as follows:

- The design of an early fusion approach based on image moments, which allows access to high-level descriptions of the initial and final scenes for all the neural layers;
- The design of early fusion based on multiple types of maps involving different levels of detail;
- An extensive analysis of early fusion approaches integrating maps that provide different levels of detail, extracted by means of feature points, segmentation, and grid-wise image moments; the models with early fusion are derived from two deep architectures and the comparison also includes a traditional visual servoing method;
- Evaluation of the proposed designs for experimental layouts with one or multiple objects.

This paper is organized as follows: Section 2 presents the usage of visual features in visual servoing. Section 3 unveils the design of the proposed early fusion architectures, while the results are discussed in Section 4. Section 5 is dedicated to the conclusions.

## 2. Visual Features in Visual Servoing

In order to perform the visual feedback control, our work consists of using visual information from cameras to determine the desired motion of a 6 DOF robot. For this, we integrated features that are highly recommended by traditional visual servoing approaches. We reconfigured these visual features to adequately incorporate them as information in the input neural layers. In order to obtain this, the main requirement is the transposition of this visual information into two-dimensional maps of the same sizes as the original RGB images. Thus, by using these types of features, some feature maps have been created to better illustrate the changes between the initial and final scenes and to draw attention to the regions from where CNN’s feature extractor can obtain relevant high-level features.

### 2.1. Image Segmentation Maps

A first method to extract visual features from images is established by the integration of image segmentation as input information, which has proven to be a valuable approach for enhancing the performances of a visual servoing system. For example, in [16] the authors are using the luminance of all pixels in an image as visual features instead of relying on geometric features, such as points, lines, or poses. Specifically, they used the grayscale intensity value of each pixel as the luminance feature in order to compute an optimization-based control law that minimizes the difference between the current and a desired image. By using the luminance of all pixels in the image, the authors avoid the need for feature extraction, which can be computationally expensive and error-prone.

**Figure 1:**
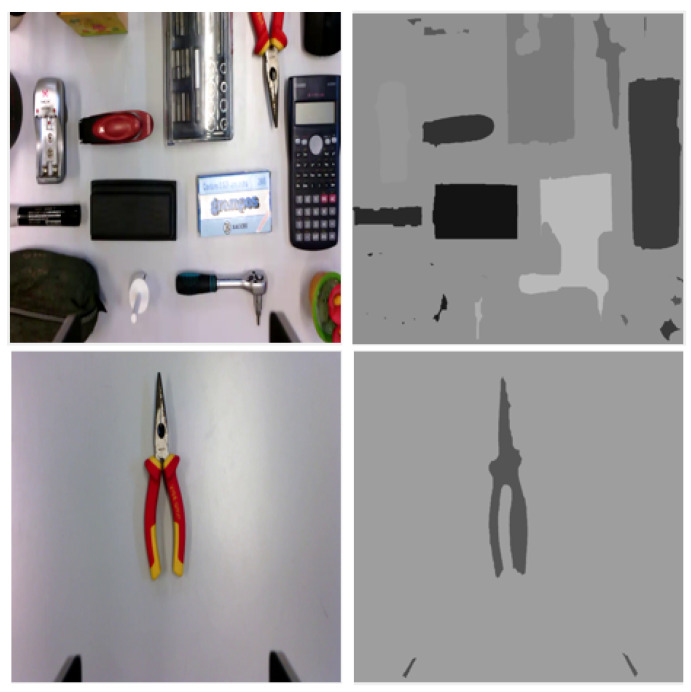
Original image (**left**) and segmented map (**right**) for complex (**up**) and simple (**down**) scenes.

Our work proposes the usage of region-based segmentation maps in which each region belonging to the background or objects is labeled with their mean intensity. The location and shape of objects can be implicitly inferred from these maps, as demonstrated in Figure 1. The resulting segmented map provides a simplified layout of the scene that can guide the feature extractor. As segmentation is region-based, it can be applied to simple or complex scenes, with uniform or non-uniform backgrounds. The usage of the mean intensities of the regions, such as labels, offers several advantages: (i) mean intensity is a relevant feature for a region, despite lighting disturbances; (ii) object matching can be implicitly ensured by the labels when the initial and final layouts have different object poses; (iii) the range of the values obtained in the segmented maps is similar to that of RGB images, making it easy for the CNNs to integrate information from multiple input channels.

Algorithm 1 outlines the main steps of the region-based segmentation algorithm that was used. Since the input images are in RGB format, a conversion to grayscale is required for the image processing steps. As Figure 1 shows, the background mainly consists of lighter areas. Therefore, computing the image complement is an important step in distinguishing between background and objects. Once a better partition is made, basic segmentation techniques such as Prewitt edge identification and morphological operations can be applied.
**Algorithm 1** Segmentation algorithm.**Require:** RGB image *I* (e.g., $I_{1}$ or $I_{2}$) 1: Convert *I* to grayscale to obtain $I_{g}$. 2: Apply image complement to $I_{g}$, in order to obtain $I_{c}$. 3: Using a disk of size $s_{o}$, apply morphological opening to $I_{c}$, to obtain $I_{o}$. 4: Subtract $I_{o}$ from $I_{c}$, to obtain $I_{w}$. Adjust the intensities of $I_{w}$. 5: Compute $I_{b}$ by extracting the edges of $I_{w}$ (Prewitt). 6: Improve $I_{b}$: perform morphological closing using a disk of size $s_{c}$, fill the holes, and erode the image using a disk of size $s_{e}$. 7: Obtain *A* by labeling the objects and background of $I_{b}$ with the corresponding mean intensity from $I_{g}$.**Ensure:** Segmented map, *A* (e.g., $A_{1}$ or $A_{2}$)

External factors, such as changes in illumination, can affect the accuracy of segmentation results. In response to these challenges, segmented maps can be used in conjunction with RGB images of the initial and final scene layouts to enable a convolutional neural network to recover crucial information from the original images. This additional information can be used to mitigate segmentation errors, refine boundary detection, and eliminate false detections. Moreover, the combined use of segmented maps and RGB images provides a more comprehensive representation of the scene, which can enhance the overall CNN performance.

Other possible solutions that could be considered for creating segmentation maps involve the usage of binary segmented maps, but this method only locates the object, without differentiating between them. Also, in the case of a non-uniform background, segmentation maps could be created by merging adjacent superpixels that share similar color properties.

### 2.2. Feature Point Maps

The second method that proposes the integration of additional information into the input array of a convolutional neural network in order to obtain more relevant features is based on interest point operators. Interest point operators are used to detect key locations from the image. These operators are specifically chosen to be less sensitive to factors such as scaling, rotation, and image quality disturbances. These features are typically extracted from images captured by a camera and used to compute the error between the current position and the desired position of the robot relative to the object or feature it is tracking. Previous works relied on the analysis of different point detectors in order to extract distinctive characteristics of an image and use them to estimate the motion of a robot. For example, in [17], the authors propose a visual servoing approach that uses SIFT feature points to track a moving object. The approach is based on a camera mounted on an anthropomorphic manipulator and the goal is to maintain a desired relative pose between the camera and the objects of interest. The papers show that the SIFT points are invariant to scale and rotation and can be used to track the object as the endpoint moves along a trajectory. The remaining robust feature points provide epipolar geometry, which is used to retrieve the motion of the camera and determine the robot’s joint angle vector using inverse kinematics. Another approach is presented in [18] where the authors propose a method for object tracking based on SURF local feature points. The visual servo controller uses geometrical features computed from the set of interest points, making the method robust against occlusion and changes in viewpoint. The experimental results were demonstrated with a robotic arm and monocular eye-in-hand camera in a cluttered environment.

In this work, we investigate the impact of early fusion on the performance of two different point operators, namely SURF (speed-up robust features) [19] and BRISK (binary robust invariant scalable key points) [20]. These two operators are selected due to their proven high performance and robustness to noise and fast computation.

By utilizing a map of the detected SURF or BRISK points, we can extract information about the location of objects in the scene. The SURF algorithm approximates the Hessian matrix by computing integral images, while the BRISK method relies on circular sampling patterns to form a binary descriptor. Both methods provide additional information about the variation of interest points between the initial and final scene layouts, which can improve CNN’s ability to understand the differences between those two point operators.

**Figure 2:**
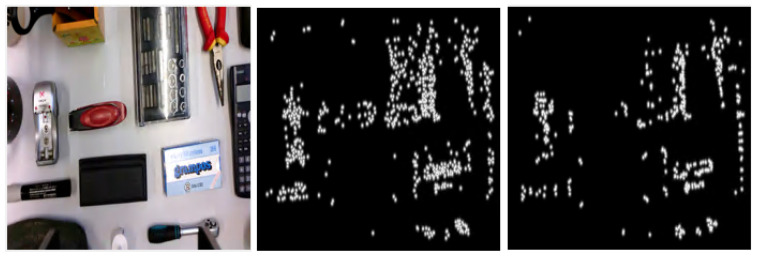
Original image (**left**) and corresponding extra map—with SURF (**middle**) and BRISK (**right**) detectors.

Algorithm 2 outlines the main steps used to configure the additional maps, where the detected points have higher gray-level intensities than the rest of the pixels. To detect the feature points, the input RGB images are first converted to grayscale and a pre-implemented algorithm for SURF or BRISK point detection is applied. These points are marked into bidimensional maps compatible with the proposed early fusion template, hence their neighborhoods are delimited by a Gaussian filter of size $f_{s}$ and standard deviation $f_{s}{}_{d}$. Finally, the resulting map is generated by mixing the neighborhoods marked around all the feature points. Examples of SURF and BRISK detectors for a scene with multiple objects are indicated in Figure 2. The maps were obtained with a Gaussian filter of size $f_{s}$ = 73 (which is about 10% of the minimum dimension of the original images) and a standard deviation of size $f_{s}{}_{d}$ = 5. This additional information can guide CNN to better understand the differences between the initial and final layouts and improve the overall accuracy of the regression task.
**Algorithm 2** Mapping using the SURF or BRISK point algorithms.**Require:** RGB image *I* (e.g., $I_{1}$ or $I_{2}$) 1: Convert *I* to grayscale, resulting in $I_{g}$. 2: For $I_{g}$, detect the features points, $p_{i}$, with i=1,…,n. 3: Create a Gaussian kernel, *K*, of size $f_{s}$, with standard deviation $f_{s}{}_{d}$. 4: For each point of interest, $p_{i}$, create a map $I_{gi}$ having the same size as $I_{g}$; $I_{gi}$ has non-zero elements only around $p_{i}$, and the neighborhood of $p_{i}$ is defined by the kernel *K* centered in $p_{i}$. 5: For each pixel (x,y) in $I_{g}$, create *A* of the same size by using the maximum value from the previously generated maps: $A\left(x,y\right)=max_{i=1,\ldots ,n}I_{gi}\left(x,y\right)$.**Ensure:** Map of feature points, *A* (e.g., $A_{1}$ or $A_{2}$)

### 2.3. Image Moment Maps

The third concept that this work uses in order to extract relevant features from the images involves image moments. Image moments are a set of mathematical properties that can be used to describe various characteristics of an image. These properties are computed from the pixel values of the image and can be used to extract features, such as the position, orientation, and shape of an object in the image. Image moments can be computed for any binary or grayscale image. In [21], the authors address this problem by establishing the analytical form of the interaction matrix for any image moment that can be computed from segmented images. They applied this approach to some basic geometric shapes and selected six combinations of moments to control the six degrees of freedom of an image-based visual servoing system. In comparison, in [22], the authors build upon the work in [21] but explore the use of both point-based and region-based image moments for visual servoing of planar objects. The authors use point-based moments to estimate the pose of the object and region-based moments to refine the pose estimate. They propose a control scheme that incorporates both types of moments and use experimental results to demonstrate the effectiveness of the approach.

Assuming that an object in an image is defined by a set of *n* points of coordinates (x,y), the image moments $m_{ij}$ of order (i+j) are defined as follows:

(1) $$m_{ij}=\sum_{k=1}^{n}x_{k}^{i}y_{k}^{i},$$

while the centered moments $\mu_{ij}$ of order (i+j) are given by:

(2) $$\mu_{ij}=\sum_{k=1}^{n}{\left(x_{k}-x_{g}\right)}^{i}{\left(y_{k}-y_{g}\right)}^{i}.$$

The $x_{g}$ = $\frac{m_{10}}{m_{00}}$ and $y_{g}$ = $\frac{m_{01}}{m_{00}}$ represent the coordinates of the gravity center, and $m_{00}$ is defined as *a*, the area of the object. The advantage of the centered moments is the invariance of the translation movements. Many other methods have been developed to find moments that are invariant to scaling and rotation, among the most well-known are Hu moments [23] and Zernike moments [24].

Apart from this, another important aspect is to ensure a correct correspondence of visual features between two successive images. For that, two well-known sets of image moments are defined by Chaumette in [21] and by Tahri and Chaumette in [22], which were also implemented in this work. The first one, proposed by Chaumette in [21], states that a feature vector could be defined as $S=(x_{g},y_{g},a,s_{x},s_{y},\alpha )$, where $x_{g}$ and $y_{g}$ are the coordinates of the gravity center and *a* is the area of an object in the image, all three considered as linear moments. The last three values of the angular moments are defined as follows:

(3) $$s_{x}=\left(c_{2}c_{3}+s_{2}s_{3}\right)/K,$$

(4) $$s_{y}=\left(s_{2}c_{3}+c_{2}s_{3}\right)/K$$

where, $c_{3}=c_{1}^{2}-s_{1}^{2}$, $s_{3}=2s_{1}c_{1}$ and $K=I_{1}I_{3}^{3/2}/\sqrt{a}$, with $I_{1}=c_{1}^{2}+s_{1}^{2}$ and $I_{3}=\mu_{20}+\mu_{02}$. The variables $c_{1},c_{2},s_{1}$, and $s_{2}$ are defined by the centered moments from (2): $c_{1}=\mu_{20}-\mu_{02}$, $c_{2}=\mu_{03}-3\mu_{21}$, $s_{1}=2\mu_{11}$ and $s_{2}=\mu_{30}-3\mu_{12}$. The sixth component, α, is defined as:

(5) $$\alpha =\frac{1}{2}\arctan\frac{2\mu_{11}}{\mu_{20}-\mu_{02}}.$$

Considering the velocity of the camera denoted with $v_{c}$ = ${\left[v,\omega \right]}^{T}$, where $v={\left[v_{x},v_{y},v_{z}\right]}^{T}$ are the linear velocities and $\omega ={\left[\omega_{x},\omega_{y},\omega_{z}\right]}^{T}$ are the angular velocities, both used to control the movement of the robot from a current configuration to a desired configuration. The linear camera velocities are controlled using the first three components of *S*, while the angular camera velocities are controlled using the last three components of *S*.

The second set of image moments considered in this work was obtained according to the equations proposed by Tahri and Chaumette in [22]. The image moments are defined by the feature vector $f_{m}$$=(x_{n},y_{n},a_{n},\tau ,\xi ,\alpha )$. The first three components are used to control the linear camera velocities and are defined as follows:

(6) $$a_{n}=Z^{*}\sqrt{\frac{a^{*}}{a}};x_{n}=a_{n}x_{g};y_{n}=a_{n}y_{g},$$

where $x_{n}$ and $y_{n}$ represent the coordinates of the gravity center, $Z^{*}$ represents the desired depth between the desired object position and the camera, $a^{*}$ is the desired object area, and *a* is the area of the object from the current configuration. Given the fact that the area *a* represents the number of point features that characterize the object and it cannot be used as a visual feature, Tahri and Chaumette propose in [22] that it should be replaced with:

(7) $$a=\mu_{20}+\mu_{02}.$$

The last three components from $f_{m}$ are used to control the angular camera velocities. For τ and ξ, the following image moments are proposed:

(8) $$\tau =\frac{I_{n1}}{I_{n3}},\xi =\frac{I_{n2}}{I_{n3}},$$

where

(9) $$\begin{array}{cc} I_{n1} & ={\left(\mu_{50}+2\mu_{32}+\mu_{14}\right)}^{2}+{\left(\mu_{05}+2\mu_{23}+\mu_{41}\right)}^{2},\\ I_{n2} & ={\left(\mu_{50}-2\mu_{32}-3\mu_{14}\right)}^{2}+{\left(\mu_{05}-2\mu_{23}-3\mu_{41}\right)}^{2},\\ I_{n3} & ={\left(\mu_{50}-10\mu_{32}+5\mu_{14}\right)}^{2}+{\left(\mu_{05}-10\mu_{23}+5\mu_{41}\right)}^{2}. \end{array}$$

The last component that is used to control the angular velocity $\omega_{z}$ is defined by the orientation angle of an object α in the same manner as in Equation (5).

Algorithm 3 summarizes the main steps used to configure the additional maps, based on angular image moments. As mentioned earlier, image moments are only some statistical descriptors that capture information about the spatial distribution of intensity values in an image. To produce maps of the same size as the input image, the image is divided into multiple cells. Also, due to the fact that the used dataset does not provide any information about $Z^{*}$ (the desired depth between the desired object position and the camera), the additional maps were generated solely based on the computation of the angular velocities.
**Algorithm 3** Mapping using the image moment algorithms.**Require:** RGB images of the desired configuration, $I_{1}$, and for the current configuration, $I_{2}$ Step 1: Convert $I_{1}$ and $I_{2}$ to binary, resulting in $I_{1}b$ and $I_{2}b$. Step 2: Divide $I_{1}b$ and $I_{2}b$ into multiple cells, each of size $m_{\mathrm{cell}}\times n_{\mathrm{cell}}$, resulting in *k* cells for an image. Step 3: Overlap each cell with 25% pixel information from each neighbor cell. Step 4: Compute angular image moments, with either Tahri or Chaumette equations, for the pair of images ($I_{1}b$, $I_{2}b$), resulting in extra maps ($A_{1}^{4}$, $A_{1}^{5}$, $A_{1}^{6}$, $A_{2}^{4}$, $A_{2}^{5}$, and $A_{2}^{6}$). Step 5: Compute all minimum and maximum values for all resulting image moment maps from Step 4. Step 6: For all image moments, perform normalization using Equation (10).**Ensure:** Map of image moments for the desired and current configurations: ($A_{1}^{4}$, $A_{1}^{5}$, $A_{1}^{6}$, $A_{2}^{4}$, $A_{2}^{5}$, and $A_{2}^{6}$)


The first step to creating such maps based on image moments consists of the conversion from RGB images to binary, using—by default—the threshold given by Otsu’s algorithm. After that, in order to compute the angular image moments either with the Tahri [22] or Chaumette [21] approach, the images were divided into equal cells of size *$m_{cell}$ × $n_{cell}$*, resulting in *k* cells for each image. The purpose is to give a more localized and detailed representation of the image moments. This is also helpful for obtaining a representation as a map, which can be integrated into the neural input array to be further processed by all convolutional layers. The next step of the algorithm consists of expanding the resulting cells to overlap with 25% pixels from each neighbor cell. Lastly, using the extended cells, six image moment maps are computed, with the following significance:

- $A_{1}^{4}$ and $A_{2}^{4}$ are the maps representing the first angular image moments, either for the desired or the current configuration scene;
- $A_{1}^{5}$ and $A_{2}^{5}$ are the maps representing the second angular image moments, either for the desired or the current configuration scene;
- $A_{1}^{6}$ and $A_{2}^{6}$ are the maps representing the third angular image moments, either for the desired or the current configuration scene.

The influence of the number of cells and the size of a cell will be discussed in the next section.

After dividing the image into cells and performing the image moments, the values of the maps were negative or larger than 255. This is problematic because images are typically represented as arrays of pixel values that range from 0 to 255. Therefore, if the image moment maps are not in this range, they alter the impact of the transfer of learning. To address this issue, a normalization step was necessary, using the minimum and maximum values of all the computed image moments, with the following equations:

(10) $$\begin{array}{cc} IM_{new} & =\left(IM_{old}-minVal_{IM}\right)/\left(maxVal_{IM}-minVal_{IM}\right)\cdot 255 \end{array}$$

where, IM can be exemplified, as stated in Algorithm 3, with one of the following image moment maps: ($A_{1}^{4}$, $A_{1}^{5}$, $A_{1}^{6}$, $A_{2}^{4}$, $A_{2}^{5}$, $A_{2}^{6}$). For example, $A_{1}^{4}{}_{n}ew$ is the normalized image moment map, $A_{1}^{4}$${}_{old}$ is the image moment map before the normalization, and $minVal_{A_{1}^{4}}$ and $maxVal_{A_{1}^{4}}$ are the minimum and maximum values of all $A_{1}^{4}$${}_{old}$ computed for the original training samples, respectively.

**Figure 3:**
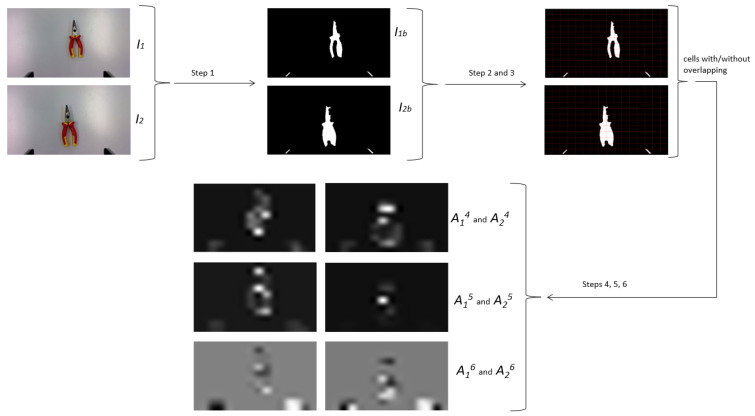
Workflow of computing angular image moments, as shown in Algorithm 3.

Figure 3 exemplifies all the steps described in Algorithm 3 for a pair of desired and current images corresponding to a simple experimental scene with a single object.

## 3. Early Fusion Architectures for Visual Servoing

In contrast to existing methods that primarily rely on hand-engineered features or geometric calculations, our proposed framework introduces a novel approach to enhancing visual feedback control through early fusion. The novelty of our approach lies in the strategic integration of additional maps alongside RGB images, a departure from the traditional reliance on visual data alone. The innovative combination not only empowers our neural network with a richer contextual understanding of the scene but also ushers in the potential for greater robustness and precision in a certain visual servoing task.

By using the concept of early fusion, we aim to capitalize on the synergy between various forms of contextual information. These additional maps, whether derived from segmentation, feature points, or image moments, are seamlessly combined with the RGB images. This approach goes beyond the typical use of pre-processed information and could empower the model to inherently learn the relationships between visual features and control commands, allowing for a more comprehensive and accurate control mechanism.

**Figure 4:**
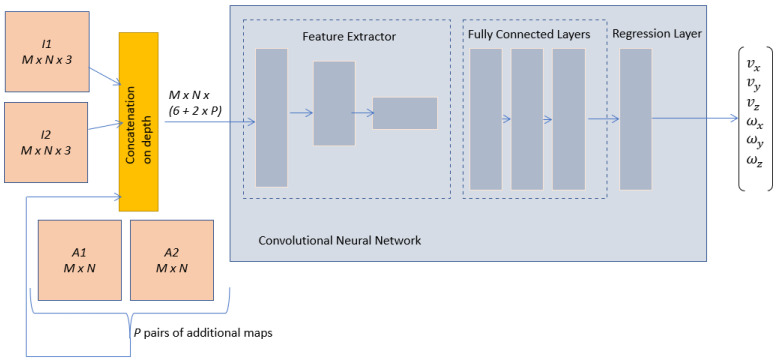
CNN general architecture using input data fusion.

The proposed network architecture is presented in Figure 4, where the input arrays are generated by concatenating the following arrays:

- $I_{1}$, of size M×N×3, is an RGB image representing the initial configuration of the scene;
- $I_{2}$, of size M×N×3, is an RGB image that describes the desired configuration of the scene;
- $A_{1}$ and $A_{2}$, both of size M×N, are additional maps that can provide supplementary important information for the learning process extracted from $I_{1}$ and $I_{2}$, respectively.

As described in Section 2, additional maps could be represented by either segmentation maps [13], points of interest [14], image moments, or their combinations, in order to improve the performances of a visual servoing neural architecture. The resulting array will be of size M×N×(6+2×P), due to the fact that the concatenation is performed on depth, where *P* represents the total depth sizes of all additional maps extracted for the initial or final image.

While it is possible to directly extract feature maps equivalent to $A_{1}$ and $A_{2}$ from $I_{1}$ and $I_{2}$ using their respective convolutional and pooling layers, including these maps as inputs to the CNN, allows the information to be effectively processed by all the neural layers. This approach also allows CNN to focus on the most significant parts of the scene while extracting deep features. For this early fusion approach, robustness against potential errors—whether from segmentation, feature points, or image moments—is ensured by the inclusion of the original images, $I_{1}$ and $I_{2}$, in the input arrays. This allows the CNN to extract any necessary information directly from them.

The input arrays with increased depth are fed into the feature extraction block, which is made up of convolutional and pooling layers. The purpose of this particular block is to create a concise description of the neural input, thereby enabling easier calculation of the linear and angular velocities. The specific design of the feature extractor and fully connected block is flexible, as long as it is suited to the size of the neural input and output. One common approach is to start with a pre-trained CNN model that has been trained for image classification and modify its architecture for the regression task. In this work, the well-known AlexNet will be such an example and modifications will be made in the last layers to make the architecture compatible with the regression task. Depending on the nature of the additional maps, different modifications must also be made in the first convolutional layer, to adjust the depth of the convolutional filters to an increased number of input channels.

One of the notable promises of our framework lies in its potential for real-time exploitation. The modifications induced by our framework do not involve a significant increase in the complexity of the deep model but rather focus on modifications to the input arrays, which are augmented with relevant information. Improved accuracy performance is expected due to the relevance of additional input data, validated by traditional visual serving techniques. By leveraging the power of a deep model and the efficiency of early fusion, our approach could have the capability to operate seamlessly in real-world environments. The early fusion also facilitates the transfer of learning from deep models devoted to image classification, which permits fast re-training. As specified before, two methods exploit maps relying on feature points or segmentation. For both methods, two additional maps are necessary to describe the initial and final scenes. These additional maps have the same sizes as the RGB images, M×N, and pixel values between [0, 255]. As the concatenation is performed on depth, the resulting input arrays have the size M×N×8, with the mean updated on each input channel.

The first convolutional layer requires another modification because pre-trained networks, such as AlexNet, are designed for RGB input images; therefore, the first convolutional layer includes filters defined for three input channels, $W_{F\times F\times 3}$, where *F* is the size of the filters. The filters of the new architecture, $W_{F\times F\times 8}^{*}$, can be initialized using the filters of the pre-trained CNN, but the initialization procedure should take into account the significance of the extra maps, to allow an effective transfer of learning. The weights of the input channels linked with $I_{1}$ and $I_{2}$ can be set to $W_{F\times F\times 3}$, while for the other two input channels, either obtained by segmentation maps or by feature point maps, their weights can be obtained from $W_{F\times F\times 3}$, using the conversion from RGB to grayscale, as the segmented maps and feature point maps are generated in grayscale:

(11) $$\begin{array}{c} W_{F}^{*}=0.299W_{R}+0.587W_{G}+0.114W_{B}. \end{array}$$

In (11), $W_{F}^{*}$ are the weights for the new filter, obtained by combining the weights from the pre-trained filter $W_{F\times F\times 3}$, which correspond to the RGB channels, namely $W_{R}$, $W_{G}$ and $W_{B}$. During the training phase, these weights will be adjusted to align with the regression task objective.

In the fully connected block, all fully connected layers from the pre-trained model can be reused, except for the last layer, which should have six neurons, one for each desired camera velocity that needs to be approximated by the model. Additionally, the activation function of the last layer should be compatible with the range of outputs, avoiding the use of activation functions, such as a rectified linear unit, which does not permit negative output values. As exemplification, the experimental section will consider the integration of AlexNet neural layers into the architecture proposed in Figure 4, resulting in three different early fusion neural architectures, based on segmentation maps, SURF feature points, and on BRISK feature points.

**Figure 5:**
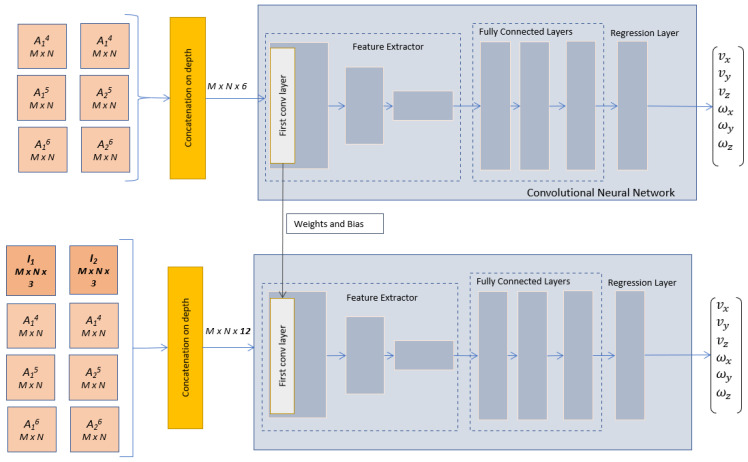
Early fusion CNN based on angular image moment maps.

A novel method proposed in this paper is based on the early fusion with image moment maps, which is described in Figure 5. Firstly, an AlexNet-based architecture is trained using the depth concatenation of the angular image moment maps, as derived from (10), with the mean updated for each input channel. The first convolutional layer includes filters with 6 input channels, one for each image moment map. These filters can be also initialized using the filters of the pre-trained AlexNet. After the training stage, the weights and bias from the first convolutional layer will be transferred to the main early fusion angular image moments-based architecture (Figure 5, bottom). The maps with the angular image moments and the original RGB images are concatenated on depth, resulting in an array of size M×N×12. Therefore, the first convolutional layer will consist of filters with 12 input channels, with the first 6 input channels linked to $I_{1}$ and $I_{2}$ initialized from the pre-trained AlexNet. The remaining 6 input channels are linked to the image moment maps and initialized from the network trained for the angular image moment maps (Figure 5, top). Although the training process might appear intricate, the model maintains its simplicity by exclusively operating at the input neural layer. We have refrained from increasing the number of layers or making them more complex. By this, the fusion of contextual information at an early stage not only enhances performance but also holds the promise of reduced computational resources during runtime utilization. This holds particular promise for applications that require rapid and continuous visual feedback control, where computational efficiency is a key factor. As an exemplification, the experimental section will consider the following comparisons regarding the angular image moment maps:

- A comparison between the angular image moments computed with the equations from [21,22];
- A comparison regarding the influence of the cells’ image size, as stated in Algorithm 3;
- A comparison regarding the influence of the overlapping cells vs. non-overlapping cells;
- A comparison between the performances of a simple early fusion visual servoing architecture (based on segmentation, feature points, or image moment maps) vs. hybrid early fusion, where different types of additional maps are combined.

## 4. Results

The importance of the additional information at the input level of a neural architecture is outlined in this section by an experimental analysis performed for the approaches previously presented. Multiple neural models were developed according to Figure 4 and Figure 5, each one receiving different information into the input array. For the experimental phase, all the networks are defined for input images resized to 227×227, in line with the pre-trained AlexNet architecture that is used for exemplification.

### 4.1. Dataset and Training

**Figure 6:**
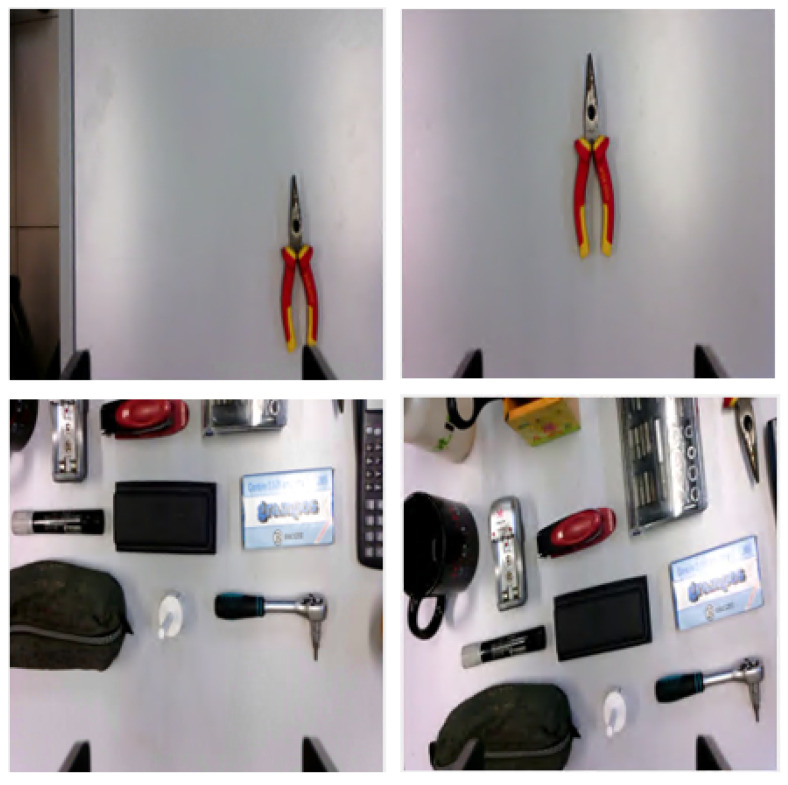
Example of dataset samples: initial (**left**) and final (**right**) layouts for experimental scenes 1 (**top**) and 2 (**bottom**).

The experimental investigations were conducted using the visual servoing dataset proposed by Ribeiro et al. [10]. As the authors of the dataset stated, the images were collected by a Kinova Gen3 robotic manipulator, in a way that approximates the self-supervised approach. The gripper’s camera, an OmniVision OV5640, acquires images of size 1280×720 at 15/30 fps. The authors from [10] programmed the robot in such a manner that it assumes different poses from a Gaussian distribution centered in the reference pose, with different standard deviations. To assess the impact of the additional maps on various input layouts, we considered two distinct configurations representing scenes of varying complexity. Specifically, one configuration featured multiple objects while the other had a single object placed against a uniform background. These two configurations were designated as experimental scenes 1 and 2 (ES1 and ES2), respectively. To provide a better understanding of the dataset, an example of the initial and final configuration is shown in Figure 6. By using this dataset, a comprehensive evaluation of the proposed method was performed, on scenes with varying complexity, in order to examine the role of the additional maps in the regression task.

For each scenario, 30.000 real-time image combinations of current and final layouts were chosen from the dataset, along with their corresponding target velocity vector. These triplets were then divided into three sets for the training, validation, and testing phases, with 70%, 15%, and 15% of the data allocated to each set, respectively. The images are disturbed by illumination variations and include foreground items that are difficult to analyze through classic visual servoing techniques. For instance, foreground regions colored similarly to the background affect the accuracy of segmentation, and areas with similar texture affect the accuracy of feature point matching.

The training process was conducted using the adaptive moment estimation (Adam) method, which is known for its efficiency. The loss function is the root mean squared error (RMSE). The training parameters were carefully selected to support effective learning. Specifically, we trained the model for 100 epochs, with an initial learning rate of 0.0001 and a mini-batch size of 64. To evaluate the performance of the trained neural models, the mean squared output error (MSE) was used, which is calculated for the training, validation, and testing samples. MSE is a well-known metric used to evaluate the performance of regression models and it measures the average of the squared differences between the predicted and actual velocity values. By analyzing the MSE values obtained for the different datasets, it was possible to gain insights into the generalization ability of the model and its capacity to accurately predict the target velocity vectors; (12) outlines the MSE computed on all output channels, for a dataset used for training, validation, or testing:

(12) $$MSE=\frac{1}{6S}\sum_{k=1}^{S}\sum_{c=1}^{6}{\left(y_{c}^{t}\left(k\right)-y_{c}^{net}\left(k\right)\right)}^{2},$$

where *S* is the number of samples from the dataset, $y_{c}^{t}\left(k\right)$ is the target, and $y_{c}^{net}\left(k\right)$ is the network output corresponding to the $c^{th}$ output channel, for the $k^{th}$ sample.

### 4.2. Influence of Early Fusion

#### 4.2.1. Simple Experimental Scene

Some preliminary results for the influence of region-based segmentation and feature point maps were conducted in [13,14]. Table 1 presents the MSE values computed for the testing dataset, considering the experimental scene ES1, with the choice of AlexNet as a pre-trained network. With this, the following architectures were developed, as stated in Figure 4:

- $M_{1}^{ES1}$, with region-based segmentation maps;
- $M_{2}^{ES1}$, with SURF feature points;
- $M_{3}^{ES1}$, with BRISK feature points;
- $M_{4}^{ES1}$, without any early data fusion.

The early fusion architectures based on feature points, $M_{2}^{ES1}$ and $M_{3}^{ES1}$, were designed according to Algorithm 2. For that, we used a Gaussian kernel of size $f_{s}$ = 27 (which is about 4% of the minimum dimension of the original images) and a standard deviation $f_{s}{}_{d}$ = 5, which allows marking reasonably sized regions around the feature points. The early fusion architecture based on region-based segmentation maps, $M_{1}^{ES1}$, was developed according to Algorithm 1, with the parameters $s_{o}$ = 100, $s_{c}$ = 10 and $s_{e}$ = 8.

As an ablation study, in this test scenario, we considered a comparison between the models with and without early fusion and a comparison between two feature point detectors, BRISK and SURF. In Table 1, the best result is outlined in green, and the baseline model without early fusion is highlighted in gray. The experiments from Table 1 show that the extra maps help CNN to focus on meaningful details. Better results were obtained with the feature point maps, due to the fact that they provide low-level details, which clearly describe the posture of the object. In contrast, the region-based segmentation maps divide the image into regions, which can be a useful way of separating the image into meaningful parts; however, because a single object is visible, these maps may not capture all the relevant information needed and potential segmentation errors become more influential. A comparison can be made between any of the models with early fusion and the baseline model, $M_{4}^{ES1}$, which has as input information only the desired and current configurations. From the perspective of MSE computed values, the early fusion-based models have better results, outlining the benefits of the proposed framework.

According to Algorithm 3 and Figure 5, Table 2 was computed as the first test scenario regarding the influence of image moment maps. With this, the following architectures were developed:

- $M_{5}^{ES1}$ is trained on angular image moments without RGB images (as stated in Figure 5—top), with *k* = 192 cells, each of size 60×80, with overlap, based on angular image moment equations from [21];
- $M_{6}^{ES1}$ is trained on the concatenation of RGB images and angular image moments (as stated in Figure 5—bottom), with *k* = 192 cells, each of size 60×80, with overlap, based on angular image moment equations from [21];
- $M_{7}^{ES1}$ is trained on angular image moments without RGB images (as stated in Figure 5—top), with *k* = 192 cells, each of size 60×80, with overlap, based on angular image moment equations from [22];
- $M_{8}^{ES1}$ is trained on the concatenation of RGB images and angular image moments (as stated in Figure 5—bottom), with *k* = 192 cells, each of size 60×80, with overlap, based on angular image moment equations from [22].

In Table 2, the best results obtained in this test scenario are outlined in green, and the baseline model from which the architectures were designed is marked in gray. As an ablation study, we considered a comparison between the origin of the angular image moment equations, either from [21,22]. Comparing the MSE values computed from Table 1 and Table 2 shows that a more accurate approximation was obtained with feature points and segmentation maps rather than image moment maps. One explanation could be that we used only angular image moments due to the fact that in the dataset [10] the depth information $Z^{*}$ was not available, but also due to the fact that image moments provide a compact representation of the shapes of an object, rather than the feature points or region-based segmentation maps which can capture a wider range of image features, such as meaningful parts from the image. Also, the early fusion with image moment maps involves using models with more learnable parameters than in the case of region-based segmentation maps and feature point maps, and this makes the training task more difficult. These two explanations are why models $M_{6}^{ES1}$ and $M_{8}^{ES1}$ do not perform better than the baseline model $M_{4}^{ES1}$, even if there is additional information at the neural input level.

The most accurate model using image moment maps, $M_{8}^{ES1}$, was configured with 192 cells with overlapping and [22] angular image moment equations. Given the fact that this model is also the best for two-stage training, two distinct configurations were also considered: one with the same number of cells defined without overlapping, and one with a higher number of cells, defined with overlapping and smaller cells. The following second scenario was considered for experimental scene 1:

- $M_{9}^{ES1}$ is trained on angular image moments without RGB images (as stated in Figure 5—top), with *k* = 192 cells, each of size 60×80, without overlap;
- $M_{10}^{ES1}$ is trained on angular image moments, with the concatenation of RGB images and angular image moments (as stated in Figure 5—bottom), with *k* = 192 cells, each of size 60×80, without overlap;
- $M_{11}^{ES1}$ is trained on angular image moments without RGB images (as stated in Figure 5—top), with *k* = 960 cells, each of size 30×32, with overlap;
- $M_{12}^{ES1}$ is trained on angular image moments, with the concatenation of RGB images and angular image moments (as stated in Figure 5—bottom), with *k* = 960 cells, each of size 30×32, with overlap.

To extend our analysis, for this ablation study we considered a comparison between a model with and without cell overlapping and between a model with a smaller number of cells ( *k* = 192 cells) and a higher number of cells (*k* = 960 cells). The MSE values are computed in Table 3, where green represents the best results obtained in this test scenario, and gray is the baseline model from which the architectures were designed.

Analyzing $M_{9}^{ES1}$ with $M_{10}^{ES1}$ or $M_{11}^{ES1}$ with $M_{12}^{ES1}$ it can be observed that the two-stage training described by Figure 5 improves the MSE values in comparison with a training stage with only the angular image moment maps. Also, a comparison can be made between $M_{10}^{ES1}$ and $M_{12}^{ES1}$ which outlines the benefits of overlapping cells (given the fact that $M_{10}^{ES1}$ was designed without overlapping cells) and also the idea that more cells of a smaller size (in this scenario 960 cells of size 30×32) are more advantageous that fewer cells (192 cells of size 60×80). A reason for that could be the fact that in the first case the 960 cells capture more fine-grained information, each cell focusing on a smaller region, allowing for better localization of image features. Given the fact that dense disparity maps were not available in the dataset and, the image moments imply using more learnable parameters while inducing data redundancy, the baseline model $M_{4}^{ES1}$ performs better but the errors are marginal in comparison with the models based on angular image moments.

#### 4.2.2. Complex Experimental Scene

Similarly to the previous subsection, we designed the neural architectures for ES2, using the methods previously described. Therefore, Table 4 presents the MSE computed for the models that integrate at the input neural layer different types of maps, each one with a different design:

- $M_{1}^{ES2}$, with region-based segmentation maps;
- $M_{2}^{ES2}$, with binary segmentation maps;
- $M_{3}^{ES2}$, with segmentation maps that are disturbed by dilation;
- $M_{4}^{ES2}$, with segmentation map that are disturbed by erosion;
- $M_{5}^{ES2}$, with SURF feature points and neighbourhoods defined by a Gaussian kernel of size $f_{s}$ = 73 and a standard deviation $f_{s}{}_{d}$ = 5;
- $M_{6}^{ES2}$, with SURF feature points and neighbourhoods defined by a Gaussian kernel of size $f_{s}$ = 73 and a standard deviation $f_{s}{}_{d}$ = 15;
- $M_{7}^{ES2}$, with BRISK feature points and neighbourhoods defined with a Gaussian kernel of size $f_{s}$ = 73 and a standard deviation $f_{s}{}_{d}$ = 5;
- $M_{8}^{ES2}$, with BRISK feature points and neighbourhoods defined with a Gaussian kernel of size $f_{s}$ = 73 and a standard deviation $f_{s}{}_{d}$ = 15;
- $M_{9}^{ES2}$, without any early data fusion.

Therefore, this ablation study allows the following analysis:

- comparison between region-based segmentation maps and binary segmentation maps, the last one meaning that each object in the image is segmented with white and the background with black, without any distinction between the foreground items;
- the sensitivity to segmentation errors that might affect the segmented maps integrated into the input arrays; the disturbed objects are dilated or eroded using a morphological square structuring element of maximum size 7 (which is about 3 % of the image size).
- comparison between two feature point detectors, BRISK and SURF, for each using two standard deviation values to highlight the neighbor pixels $f_{s}{}_{d}$.

For the early fusion based on segmentation, a comparison between $M_{1}^{ES2}$ and $M_{2}^{ES2}$ show that region-based segmentation maps are more valuable than the binary maps. Because the binary maps just locate the objects, without differentiating between them, the early fusion provides less extra information and consequently has a reduced impact on the CNN performance. The sensitivity to segmentation errors implies a comparison between $M_{1}^{ES2}$, $M_{3}^{ES2}$ and $M_{4}^{ES2}$. According to this, the errors between the models working with disturbed and non-disturbed maps are marginal. The explication is related to the fact that CNNs can also exploit the information provided by the original images to correct some segmentation errors. This result is important to highlight that input data redundancy produced by early fusion can be also exploited to improve the robustness of the model.

The last aspect implied in this ablation study involves a comparison between models working with feature point maps. The experiments were conducted for two detectors, BRISK and SURF, using two different standard deviations to investigate whether it is more advantageous to delineate larger or smaller neighbourhoods around the feature points. A larger neighbourhood can be beneficial if the detector does not yield enough key points, while smaller neighbourhoods are valuable for narrowing down the recommended exploration area when all required key points have been adequately identified. The results show that both BRISK and SURF detectors identify noteworthy points of interest. However, as indicated in Table 4, the SURF points tend to be more relevant, on average, leading to much lower MSE values for $M_{5}^{ES2}$ and $M_{6}^{ES2}$ vs. $M_{7}^{ES2}$ and $M_{8}^{ES2}$. The results also indicate that the choice of the detector has a greater impact than the size of the neighbourhood defined around the points of interest. The differences between $M_{5}^{ES2}$ vs. $M_{6}^{ES2}$ and $M_{7}^{ES2}$ vs. $M_{8}^{ES2}$ are minor, even though the maps account for neighbourhoods of varying sizes.

Also, a comparison can be made between any of the models built via the framework from Figure 4 and the baseline model, $M_{9}^{ES2}$, which has as input information only the desired and current configurations. Analyzing the MSE values, any model with early fusion has better results, a fact that outlines the benefits of the proposed framework. In comparison with the results presented in Table 1, the segmentation maps are more influential for the neural architecture vs. the feature point maps. Also, it seems that the feature points are more relevant in the simple experimental scene because they capture the unique characteristics of the single object in the scene, allowing the neural network to recognize it easily. On the other hand, in the more complex scene with multiple objects, the feature points do not directly differentiate between objects. Segmentation maps may be more effective because they provide a way to isolate each object from the background and thus make it easier for the neural network to distinguish between them.

As in the simple experimental scene, two different scenarios were considered for designing the angular image moments into the first neural architecture. For our ablation study we considered the comparison between the two techniques of defining the angular image moments, either with the equations from [21,22]. The resulting neural configurations are described as follows, with the MSE computed in Table 5:

- $M_{10}^{ES2}$ is trained on angular image moments without RGB images (as stated in Figure 5—top), with *k* = 192 cells, each of size 60×80, with overlap, based on angular image moment equations from [21];
- $M_{11}^{ES2}$ is trained on the concatenation of RGB images and angular image moments (as stated in Figure 5—bottom), with *k* = 192 cells, each of size 60×80, with overlap, based on angular image moment equations from [21];
- $M_{12}^{ES2}$ is trained on angular image moments without RGB images (as stated in Figure 5—top), with *k* = 192 cells, each of size 60×80, with overlap, based on angular image moment equations from [22];
- $M_{13}^{ES2}$ is trained on the concatenation of RGB images and angular image moments (as stated in Figure 5—bottom), with *k* = 192 cells, each of size 60×80, with overlap, based on angular image moment equations from [22].

In the same manner, as in the Simple experimental scene, a comparison of the MSE values can be made between Table 4 and Table 5. It results that a more accurate approximation was obtained with feature points and segmentation maps rather than with image moment maps. One explanation could be that we used only angular image moments due to the fact that in the dataset [10] the depth information $Z^{*}$ was not available, but also due to the fact that image moments provide a compact representation of the shapes of an object, rather than the feature points or region-based segmentation maps which can capture a wider range of image features, such as meaningful parts from the image. Also, the early fusion with image moment maps involves using models with more learnable parameters than in the case of region-based segmentation maps and feature point maps, and this makes the training task more difficult. The presence of these two factors also explains why models like $M_{6}^{ES1}$ and $M_{8}^{ES1}$ do not exhibit superior performance compared to the baseline model $M_{4}^{ES1}$, regardless of the inclusion of extra information in the neural input arrays.

A second test scenario was considered for the complex experimental scene, focusing on the comparison of the model with the best results from the first scenario test, $M_{13}^{ES2}$. The model was configured with Tahri’s angular image moment equations [22], with 192 cells of size 60×80 for which the following modifications were performed:

- $M_{14}^{ES2}$ is trained on angular image moments without RGB images (as stated in Figure 5—top), with *k* = 192 cells, each of size 60×80, without overlap;
- $M_{15}^{ES2}$ is trained on angular image moments, with the concatenation of RGB images and angular image moments (as stated in Figure 5—bottom), with *k* = 192 cells, each of size 60×80, without overlap;
- $M_{16}^{ES2}$ is trained on angular image moments without RGB images (as stated in Figure 5—top), with *k* = 960 cells, each of size 30×32, with overlap;
- $M_{17}^{ES2}$ is trained on angular image moments, with the concatenation of RGB images and angular image moments (as stated in Figure 5—bottom), with *k* = 960 cells, each of size 30×32, with overlap.

From these four configurations, it results as an ablation study the analysis of two different cell sizes and the design of the image moment maps with or without cell overlapping. Table 6 showcases the computed Mean Squared Error (MSE) values for the four models under consideration. When examining $M_{14}^{ES2}$ alongside $M_{15}^{ES2}$ or $M_{16}^{ES2}$ alongside $M_{17}^{ES2}$, it can be observed that employing the two-stage training approach depicted in Figure 5 leads to superior MSE values compared to single-stage training using only the angular image moment maps.

Additionally, a comparison between $M_{15}^{ES2}$ and $M_{17}^{ES2}$ highlights the advantages of incorporating overlapping cells (considering that $M_{15}^{ES2}$ was designed without overlapping cells). Moreover, the concept of having more cells of a smaller size (in this scenario, 960 cells of size 30×32) proves to be more advantageous than having fewer cells (192 cells of size 60×80). One potential reason for this lies in the fact that in the former case, the 960 cells capture finer details and information, with each cell focusing on a smaller region. Consequently, this facilitates a more precise localization of image features. In the absence of dense disparity maps and using image moments, which implies more learnable parameters, the baseline model $M_{9}^{ES2}$ performs better but the errors are marginal in comparison with the models based on angular image moments. These results highlight that reducing the number of additional input maps is essential for an effective early fusion, as each extra map introduces data redundancy and demands increasing the depth of the filters from the first convolutional layer.

#### 4.2.3. Influence of Hybrid Maps

Regarding our visual servoing task, we considered feature points as low-level information, segmentation regions as mid-level information, and angular image moments as high-level information. As observed in Section 4.2.1 and Section 4.2.2, an architecture with high-level information could be too comprehensive for a neural network, so the additional information might not be eloquent enough in the training process. On the other hand, more compact information, such as region segmentation maps or feature points, could be more useful regarding the nature of the information from which the neural layers take valuable additional features.

Therefore, in our tests, we considered the analysis of the influence of multiple types of extra maps, meaning the concatenation of segmentation and SURF maps alongside the RGB images. We considered the best configurations of the segmentation maps ($s_{o}$ = 100, $s_{c}$ = 10 and $s_{e}$ = 8—According to Algorithm 1) and SURF feature maps (Gaussian kernel of size $f_{s}$ = 27 and a standard deviation $f_{s}{}_{d}$ = 5—According to Algorithm 2), resulting in an input neural array of size 227 × 227 × 10. Given the nature of the experimental scenes, two different architectures were designed and tested, with their MSE values being described in Table 7.

Analyzing the MSE values from Table 7 for experimental scene 1, $M_{13}^{ES1}$ configured with hybrid maps does not perform better than $M_{2}^{ES1}$ configured with only SURF interest point maps, but has close values with $M_{1}^{ES1}$, configured with segmentation maps. For the complex scene, the MSE values of the $M_{18}^{ES2}$ model are comparable to those of the $M_{2}^{ES2}$ model configured with SURF interest point maps, but poorer than those of the $M_{1}^{ES2}$, configured with segmentation maps. The usage of multiple types of extra maps in the neural input arrays does not bring benefits to the accuracy of the model. An explanation could be related to the fact that these hybrid early fusions increase the level of data redundancy, as well as the complexity of the model, making the training more difficult to manage.

#### 4.2.4. Discussions

To highlight the impact of early fusion, two other test scenarios were performed using another deep model as a baseline. We selected one of the models from [10] which had the best results (named by the authors Model 1—Direct Regression). This model was extended for early fusion, according to the framework from Figure 4. The results are compared with the models derived from AlexNet. Given the fact that for experimental scene 1 the best result was obtained with $M_{2}^{ES1}$, the following test scenario analysis was performed:

- $M_{2}^{ES1}$, with SURF feature points based on AlexNet;
- $M_{4}^{ES1}$, without any early data fusion based on AlexNet;
- $M_{14}^{ES1}$, with SURF feature points based on the model from [10];
- $M_{15}^{ES1}$, without any early data fusion based on the model from [10].

The MSE results are presented in Table 8; for $M_{14}^{ES1}$ and $M_{15}^{ES1}$, the trainings were performed in the same conditions as in the earlier configurations.

The results listed in Table 8 show that the models based on the baseline AlexNet perform better than those built from the model indicated in [10] ($M_{2}^{ES1}$ vs. $M_{14}^{ES1}$ and $M_{4}^{ES1}$ vs. $M_{15}^{ES1}$). The explanation could be related to the fact that, in our implementation, the parameters of $M_{14}^{ES1}$ and $M_{15}^{ES1}$ were initialised by means of the Glorot algorithm [25], without transfer of learning from a pre-trained model. On the contrary, as explained in the previous section, in the case of $M_{2}^{ES1}$ and $M_{4}^{ES1}$, the parameters were advantageously initialised via transfer of learning from the pre-trained AlexNet. As anticipated, the pre-existing knowledge from a pre-trained model proves valuable in facilitating effective training. For configurations like $M_{14}^{ES1}$ and $M_{15}^{ES1}$, it becomes evident that a more extended training phase could potentially yield benefits. Nevertheless, even within this setup, the model employing input data fusion, as stated by the framework described in Figure 4, demonstrates superior MSE values compared to the conventional baseline model utilizing only six input channels. This indicates that additional maps integrated into the input arrays were helpful for understanding the characteristics of the scenes in the framework of the visual servoing task.

In the same manner, the testing scenario for experimental scene 2 was defined starting from the best models obtained for it, namely $M_{1}^{ES2}$, which uses region-based segmentation maps:

- $M_{1}^{ES2}$, with region-based segmentation maps, based on AlexNet;
- $M_{9}^{ES2}$, without any early fusion data, based on AlexNet;
- $M_{19}^{ES2}$, with region-based segmentation maps, based on the model from [10];
- $M_{20}^{ES2}$, without any early fusion data, based on the model from [10].

The MSE results are presented in Table 9; for $M_{19}^{ES2}$ and $M_{20}^{ES2}$, the trainings were performed in the same conditions as in the earlier configurations.

For both deep models that are set as references, the segmented maps provide meaningful input data that can be effectively utilized by the neural layers ($M_{1}^{ES2}$ vs. $M_{9}^{ES2}$ and $M_{19}^{ES2}$ vs. $M_{20}^{ES2}$). Across all output channels, the MSE values are notably lower for the architectures employing early fusion. Also, the models derived from the pre-trained AlexNet outperform those constructed from the model from [10]. $M_{19}^{ES2}$ and $M_{20}^{ES2}$ are trained from scratch without any transfer of learning, beginning with the initial values supplied by the Glorot method [25]. Consequently, these models might require extended training times or larger datasets. Nevertheless, even in this initial learning phase, early fusion proves to be advantageous.

### 4.3. Control Scenario Analysis

In the regression task from this work, multiple CNNs are trained to predict velocities as outputs, which control the motion of a camera of a robotic system based on visual input. Alongside the previously computed metrics, we evaluate the best models from experimental scenes 1 and 2, by illustrating the velocities computed during the control scenario and the autocorrelation of the residuals resulting from the predicted velocities. The analysis is conducted for both experimental scenes.

To properly evaluate the performances of the proposed approaches, in the numerical analysis, we include the results generated by a classic visual servoing control law. The choice was to add PBVS to the evaluation because the dataset includes the camera’s pose-related information. PBVS considers as control features the information stored by a translation vector, **t**, and an angle–axis representation of the orientation, θu. If the translation vector is defined as the translation between the current camera frame related to the desired camera frame, ${}^{c}*t_{c}$, then considering the current features

(13) $$s=({{}^{c}*t}_{c},\theta u),$$

and the desired ones

(14) $$s^{*}=\left(0,0\right),$$

according to [1], the control law that computes the values of the linear and angular camera velocities $v_{c}={\left[v_{x}\;v_{y}\;v_{z}\;\omega_{x}\;\omega_{y}\;\omega_{z}\right]}^{T}$ results as follows:

(15) $$v_{c}=\left[\begin{array}{c} -\lambda R^{T}{{}^{c}*t}_{c}\\ -\lambda \theta u \end{array}\right],$$

where R is the rotation matrix and λ is the proportional gain.

#### 4.3.1. Velocity Visualization

**Figure 7:**
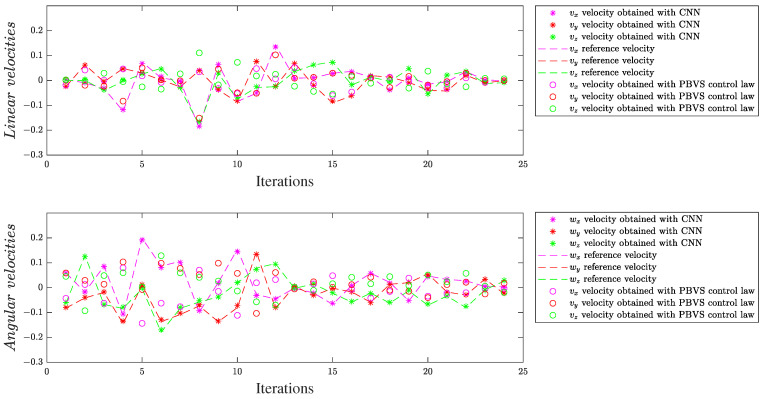
Linear (**top**) and angular (**bottom**) generated velocities for the simple experimental scene, ES1.

**Figure 8:**
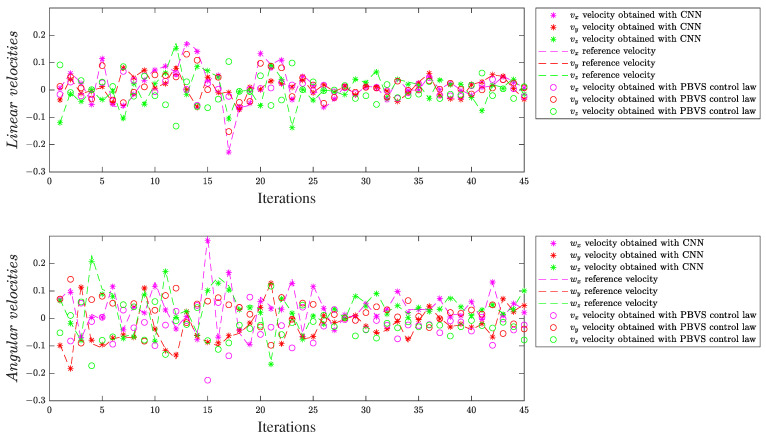
Linear (**top**) and angular (**bottom**) generated velocities for the complex experimental scene, ES2.

For the same scenario, $M_{2}^{ES1}$ for experimental scene 1 and $M_{1}^{ES2}$ for experimental scene 2, we considered multiple consecutive frames extracted from the testing dataset, where we analyzed the behaviors of the linear and angular velocities obtained by those two models in comparison with their expected target data and with the velocities obtained by the control law from (15). Some plots are exemplified in Figure 7 and Figure 8. The best performance of the PBVS control law in comparison to the reference velocities was obtained after conducting multiple tests for the proportional gain λ = 0.72. As observed, the neural models predict values closer to the references in comparison with the velocities obtained by the control law from (15). These results show that, compared to PBVS, the proposed approach can decouple all the degrees of freedom of the camera motion in relation to the visual information, not only linear velocities versus the angular velocities; this shows the benefits of the framework based on deep models with early fusion.

#### 4.3.2. Autocorrelation of Residuals

**Figure 9:**
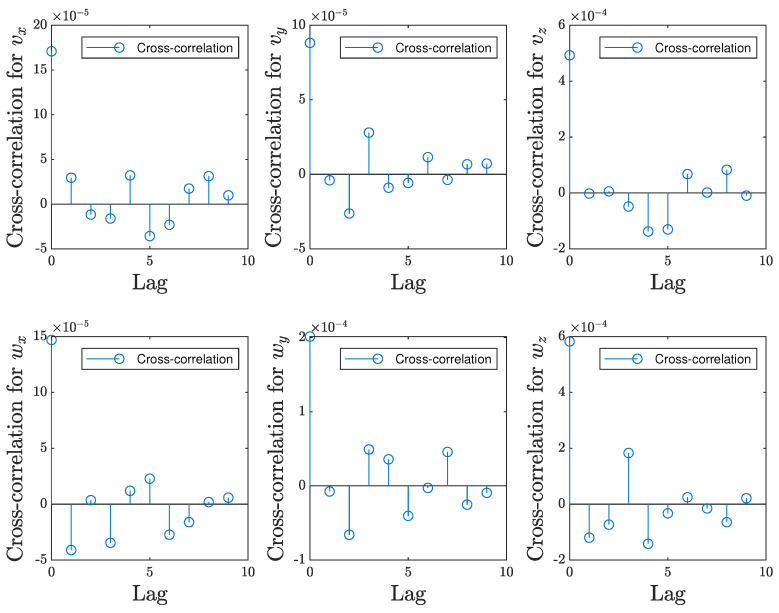
Autocorrelation of residuals for the simple experimental scene, ES1.

**Figure 10:**
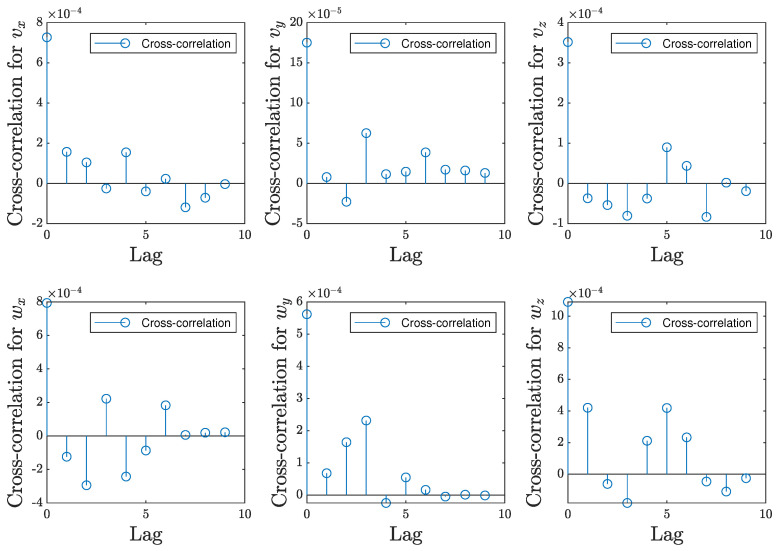
Autocorrelation of the residuals for the complex experimental scene, ES2.

The autocorrelation of residuals was analyzed for the same models, $M_{2}^{ES1}$ for experimental scene 1 and $M_{1}^{ES2}$ for experimental scene 2. The residual is computed as the error between the target and prediction; therefore, the autocorrelation of the residuals helps to identify systematic patterns or dependencies in the prediction errors at different time lags. Given the size of the testing dataset, we extracted only 10 predicted and target data for which we visually analyzed the autocorrelation, with the mention that the same distribution of the values is maintained for all the values resulting from the testing data. Therefore, Figure 9 illustrates the analysis for model $M_{2}^{ES1}$, for the experimental scene 1, with respect to all 6 velocity components predicted by the model. In the same manner, Figure 10 shows the same analysis for the model $M_{1}^{ES2}$ in experimental scene 2.

As observed in Figure 9 and Figure 10, the magnitude of autocorrelation is much higher for the lead/lag 0 than for any other lead. This indicates there is no systematic bias in the predictions, resulting in, on average, the predicted values being close to the corresponding target values. Also, in every plot pertaining to each velocity component in both Figure 9 and Figure 10, the autocorrelation values gradually approach to zero. In line with the system identification methodology, this suggests that the residuals, while highly correlated with themselves at the same time point, do not exhibit systematic trends over time. This characteristic mirrors the properties of white noise, where data points are independent and identically distributed, and there is no meaningful temporal structure.

## 5. Conclusions

This paper introduces CNN architectures with early fusion for a visual servoing task in the context of the camera positioning on a 6 DOF gripper robot. The neural input array is expanded on depth, by combining the RGB images (corresponding to the initial and final scenes) with some additional maps. The role of these maps is to provide simplified sketches of the initial and final scenes, which can guide CNN in extracting meaningful features.

Some of the most effective traditional visual servoing techniques were explored to generate extra maps with different levels of detail, relevant to the approximation of the linear and angular camera velocities required by the visual controller. This analysis focuses on the design of early fusion approaches using the following types of maps (stand-alone or in combination): angular moment maps, region-based segmented maps, and feature point maps. Each type of map offers a different level of information extracted from initial and final images. To allow simple training, the transfer of learning from a CNN pre-trained for image classification is adopted. The transfer of learning is adjusted to also manage the supplementary neural parameters from the first convolutional layer, which were introduced due to the use of early fusion.

We evaluated the deep models on two different scenes, with a single object and multiple objects, respectively. These experimental scenarios allowed investigating what level of detail is helpful for the CNN design and the limitations resulting from early fusion. Mainly, each extra map increases the level of input data redundancy and requires the use of additional neural parameters in the deep model. In this context, we concluded that low-level (SURF feature points) and mid-level information (segmentation maps) are more helpful than high-level information, such as image moments. A feature point technique and region-segmentation technique were configured to produce a single supplementary map for each image, to highlight the important areas, such that the differences between the initial and final scenes can be easily found by CNN.

Future work will focus on evaluating the performances of the proposed early fusion-based CNN architectures in applications for eye-in-hand and eye-to-hand configurations. An envisaged step involves the acquisition of images combined with dense disparity maps to allow the integration of additional maps with linear image moments. The potential for improved precision and robustness offered by the combined input information holds promise for advancing the field of visual servoing and facilitating practical applications in domains requiring precise camera positioning and control.

## Figures and Tables

**Table 1:** MSE for segmentation and feature point models—with and without early fusion—on the testing dataset of experimental scene 1.

No.	CNN	with Early Fusion?	$v_{x}$ [m/s $\times 10^{-5}$]	$v_{y}$ [m/s $\times 10^{-5}$]	$v_{z}$ [m/s $\times 10^{-5}$]	$\omega_{x}$ [${}^{\circ }$/s $\times 10^{-5}$]	$\omega_{y}$ [${}^{\circ }$/s $\times 10^{-5}$]	$\omega_{z}$ [${}^{\circ }$/s $\times 10^{-5}$]
1	$M_{1}^{ES1}$	yes	1.5920	2.2058	4.3378	3.0857	1.7223	4.4991
2	$M_{2}^{ES1}$	yes	0.8058	1.1116	2.1964	1.4215	0.8726	2.9742
3	$M_{3}^{ES1}$	yes	1.2849	1.8305	2.7937	2.0749	1.4618	3.6792
4	$M_{4}^{ES1}$	no	2.0086	2.3068	5.1554	3.4767	2.3897	6.1174

**Table 2:** MSE values for angular image moment models—first scenario—on the testing dataset of experimental scene 1.

No.	CNN	with Early Fusion?	$v_{x}$ [m/s $\times 10^{-5}$]	$v_{y}$ [m/s $\times 10^{-5}$]	$v_{z}$ [m/s $\times 10^{-5}$]	$\omega_{x}$ [${}^{\circ }$/s $\times 10^{-5}$]	$\omega_{y}$ [${}^{\circ }$/s $\times 10^{-5}$]	$\omega_{z}$ [${}^{\circ }$/s $\times 10^{-5}$]
1	$M_{5}^{ES1}$	yes	4.8836	6.2189	9.0847	15.6177	16.6287	16.2375
2	$M_{6}^{ES1}$	yes	2.2945	2.7545	5.5684	3.7191	2.5188	5.6436
3	$M_{7}^{ES1}$	yes	4.5350	6.3571	9.8342	16.1768	14.4885	16.8532
4	$M_{8}^{ES1}$	yes	2.2290	2.4258	5.3002	3.4879	2.5096	5.4063
5	$M_{4}^{ES1}$	no	2.0086	2.3068	5.1554	3.4767	2.3897	6.1174

**Table 3:** MSE values for angular image moment models—second scenario—on the testing dataset of experimental scene 1.

No.	CNN	with Early Fusion?	$v_{x}$ [m/s $\times 10^{-5}$]	$v_{y}$ [m/s $\times 10^{-5}$]	$v_{z}$ [m/s $\times 10^{-5}$]	$\omega_{x}$ [${}^{\circ }$/s $\times 10^{-5}$]	$\omega_{y}$ [${}^{\circ }$/s $\times 10^{-5}$]	$\omega_{z}$ [${}^{\circ }$/s $\times 10^{-5}$]
1	$M_{9}^{ES1}$	yes	4.7235	6.8951	9.8050	16.3303	16.4116	17.1293
2	$M_{10}^{ES1}$	yes	2.0969	2.6951	5.1780	3.9697	2.5908	5.8394
3	$M_{11}^{ES1}$	yes	4.2983	5.6004	10.0449	18.2033	13.5218	16.0985
4	$M_{12}^{ES1}$	yes	2.0795	2.5924	5.1154	3.6769	2.5534	5.6018
4	$M_{4}^{ES1}$	no	2.0086	2.3068	5.1554	3.4767	2.3897	6.1174

**Table 4:** MSE values for segmentation and feature point models with and without early fusion on the testing dataset of experimental scene 2.

No.	CNN	with Early Fusion?	$v_{x}$ [m/s $\times 10^{-5}$]	$v_{y}$ [m/s $\times 10^{-5}$]	$v_{z}$ [m/s $\times 10^{-5}$]	$\omega_{x}$ [${}^{\circ }$/s $\times 10^{-5}$]	$\omega_{y}$ [${}^{\circ }$/s $\times 10^{-5}$]	$\omega_{z}$ [${}^{\circ }$/s $\times 10^{-5}$]
1	$M_{1}^{ES2}$	yes	1.5070	1.3412	2.4003	4.2160	2.5891	4.0299
2	$M_{2}^{ES2}$	yes	2.0613	2.6963	5.1033	3.4469	2.0103	5.7278
3	$M_{3}^{ES2}$	yes	1.5887	1.3959	2.5151	4.4366	2.6301	4.3738
4	$M_{4}^{ES2}$	yes	1.5899	1.4282	2.5555	4.4581	2.8492	4.3276
5	$M_{5}^{ES2}$	yes	1.5887	1.3959	2.5151	4.4366	2.6301	4.3738
6	$M_{6}^{ES2}$	yes	1.6600	1.4653	2.2952	4.8211	3.0827	4.3262
7	$M_{7}^{ES2}$	yes	2.3339	2.1877	3.2787	7.9396	5.0341	5.5175
8	$M_{8}^{ES2}$	yes	2.3434	2.2612	3.3294	7.8815	5.0178	5.7616
9	$M_{9}^{ES2}$	no	2.9209	2.8154	4.0592	8.9356	6.3830	6.4336

**Table 5:** MSE values for angular image moment models—first scenario—on the testing dataset of experimental scene 2.

No.	CNN	with Early Fusion?	$v_{x}$ [m/s $\times 10^{-5}$]	$v_{y}$ [m/s $\times 10^{-5}$]	$v_{z}$ [m/s $\times 10^{-5}$]	$\omega_{x}$ [${}^{\circ }$/s $\times 10^{-5}$]	$\omega_{y}$ [${}^{\circ }$/s $\times 10^{-5}$]	$\omega_{z}$ [${}^{\circ }$/s $\times 10^{-5}$]
1	$M_{10}^{ES2}$	yes	5.8103	5.0114	7.0448	16.4421	15.5449	13.2508
2	$M_{11}^{ES2}$	yes	3.7898	3.4630	5.0784	12.5233	8.8326	8.4779
3	$M_{12}^{ES2}$	yes	5.5286	4.7391	6.7281	13.8621	12.7349	10.6388
4	$M_{13}^{ES2}$	yes	3.3418	2.8719	4.5878	9.9417	6.8617	7.6917
5	$M_{9}^{ES2}$	no	2.9209	2.8154	4.0592	8.9356	6.3830	6.4336

**Table 6:** MSE values for angular image moment models—second scenario—on the testing dataset of experimental scene 2.

No.	CNN	with Early Fusion?	$v_{x}$ [m/s $\times 10^{-5}$]	$v_{y}$ [m/s $\times 10^{-5}$]	$v_{z}$ [m/s $\times 10^{-5}$]	$\omega_{x}$ [${}^{\circ }$/s $\times 10^{-5}$]	$\omega_{y}$ [${}^{\circ }$/s $\times 10^{-5}$]	$\omega_{z}$ [${}^{\circ }$/s $\times 10^{-5}$]
1	$M_{14}^{ES2}$	yes	6.9171	5.7221	7.1592	13.6263	12.0160	13.4043
2	$M_{15}^{ES2}$	yes	3.0378	2.8809	4.2012	9.8314	6.6217	6.9574
3	$M_{16}^{ES2}$	yes	5.3859	4.5193	6.4891	10.5421	10.1589	9.8788
4	$M_{17}^{ES2}$	yes	3.0113	2.8598	4.1895	9.7834	6.5659	6.8982
9	$M_{9}^{ES2}$	no	2.9209	2.8154	4.0592	8.9356	6.3830	6.4336

**Table 7:** MSE values for hybrid maps on the testing dataset of experimental scene 1 and 2.

No.	CNN	with Early Fusion?	$v_{x}$ [m/s $\times 10^{-5}$]	$v_{y}$ [m/s $\times 10^{-5}$]	$v_{z}$ [m/s $\times 10^{-5}$]	$\omega_{x}$ [${}^{\circ }$/s $\times 10^{-5}$]	$\omega_{y}$ [${}^{\circ }$/s $\times 10^{-5}$]	$\omega_{z}$ [${}^{\circ }$/s $\times 10^{-5}$]
1	$M_{13}^{ES1}$	yes	1.3974	1.6353	3.3363	2.4560	1.5672	3.6322
2	$M_{18}^{ES2}$	yes	2.7728	2.6561	3.7574	9.1100	6.1642	6.69544

**Table 8:** MSE values for different deep learning models on the testing dataset of experimental scene 1.

No.	CNN	with Early Fusion?	$v_{x}$ [m/s $\times 10^{-5}$]	$v_{y}$ [m/s $\times 10^{-5}$]	$v_{z}$ [m/s $\times 10^{-5}$]	$\omega_{x}$ [${}^{\circ }$/s $\times 10^{-5}$]	$\omega_{y}$ [${}^{\circ }$/s $\times 10^{-5}$]	$\omega_{z}$ [${}^{\circ }$/s $\times 10^{-5}$]
1	$M_{2}^{ES1}$	yes	0.8058	1.1116	2.1964	1.4215	0.8726	2.9742
2	$M_{4}^{ES1}$	no	2.0086	2.3068	5.1554	3.4767	2.3897	6.1174
3	$M_{14}^{ES1}$	yes	22.7901	20.8532	23.8162	33.2861	31.1923	37.1726
4	$M_{15}^{ES1}$	no	26.3546	23.8321	26.9956	38.4817	34.2442	39.3864

**Table 9:** MSE values for different deep learning models on the testing dataset of experimental scene 2.

No.	CNN	with Early Fusion?	$v_{x}$ [m/s $\times 10^{-5}$]	$v_{y}$ [m/s $\times 10^{-5}$]	$v_{z}$ [m/s $\times 10^{-5}$]	$\omega_{x}$ [${}^{\circ }$/s $\times 10^{-5}$]	$\omega_{y}$ [${}^{\circ }$/s $\times 10^{-5}$]	$\omega_{z}$ [${}^{\circ }$/s $\times 10^{-5}$]
1	$M_{1}^{ES2}$	yes	1.5070	1.3412	2.4003	4.2160	2.5891	4.0299
2	$M_{9}^{ES2}$	no	2.9209	2.8154	4.0592	8.9356	6.3830	6.4336
3	$M_{19}^{ES2}$	yes	16.8315	15.4620	19.1508	32.1703	28.1445	32.0806
4	$M_{20}^{ES2}$	no	26.3546	23.8321	26.9956	38.4817	34.2442	39.3864

## Data Availability

The data used to support the findings of this study are available from the corresponding author upon request.
